# Developmental-behavioral pediatrics education in the United States: challenges in the midst of healthcare evolution

**DOI:** 10.5116/ijme.59f4.2cf5

**Published:** 2017-11-10

**Authors:** Neelkamal Soares, Rebecca Baum, Dilip Patel

**Affiliations:** 1 Division of Developmental-Behavioral Pediatrics, Department of Pediatric and Adolescent Medicine, Western Michigan University Homer Stryker MD School of Medicine, USA; 2 Division of Developmental-Behavioral Pediatrics, Nationwide Children's Hospital, USA

**Keywords:** Developmental-behavioral, pediatrics, education, United States, healthcare

## Introduction

Education in healthcare and delivery of healthcare in the 21^st^ century is changing, moving towards an interdisciplinary model that benefit both patients and physicians alike. An interdisciplinary approach enables coordinated and coherent linkages between disciplines resulting in reciprocal interactions that overlap disciplinary boundaries, and generate new common methods, knowledge, and perspectives.^1^ Providing interdisciplinary education is essential to produce healthcare providers with the knowledge and skills required to optimally collaborate in working environments.^2^ Due to the value of this type of education, the World Health Organization (WHO) and United States (US) Health Resources and Services Administration (HRSA) have engaged in efforts to advance interdisciplinary education.^3^^-^^5^

One of HRSA's initiatives through the Maternal and Child Health Bureau (MCHB) is focused on developing training programs for Developmental-Behavioral Pediatrics (DBP), a subspecialty of pediatrics involved in the evaluation, counseling, and provision of treatment for a wide range of developmental and behavioral concerns and conditions. This includes learning disorders and other school-related problems, attention and behavioral disorders, habit and regulatory disorders, neurodevelopmental disabilities, delayed development in various areas, as well as behavioral and developmental conditions accompanying chronic illnesses.^6^ DBP was formally recognized as a pediatric subspecialty in 1999, with two primary aims: 1) to educate trainees and general pediatricians in practice to deliver improved care for children and adolescents with developmental-behavioral concerns and 2) to expand the knowledge base related to children's developmental and behavioral problems through research. Over the last two decades, DBP, as a distinct discipline, has expanded to deliver clinical care in a variety of settings beyond academic tertiary care centers. By the end of 2016, the American Board of Pediatrics recognized almost 800 board certified Developmental-Behavioral pediatricians (DBPs),^7^ which represents a small proportion of all pediatricians (118,200) and pediatric subspecialists (28,300) in the US.

This perspective paper aims to highlight the challenges faced by DBP educators and trainees in an evolving healthcare landscape and suggest strategies to leverage these challenges to opportunities.

### Role of educators in DBP

DBPs are charged with training future general pediatricians (through pediatric residency) and the next generation of DBPs (through fellowship), as well as supporting current general pediatricians who continue to report reservations about how to properly manage common developmental and behavioral concerns.^8^ Pediatric residency and fellowship training competencies highlight the importance of systems based practice, which is an important component of interdisciplinary care.^9^ The DBP specialty embraces, understands and champions interdisciplinary practice,^10^ and DBP fellowship training has been recognized as an opportunity to prepare trainees in addressing the needs of the cognitive, affective, and behavioral health of children and adolescents using interdisciplinary team experiences.^11^ Families and children benefit from the interdisciplinary approach utilized by DBPs who provide comprehensive approaches to behavior management and advocate for families navigating complex systems of care.

DBPs have been increasingly involved in online curricular development at all levels of medical education through collaboration with governmental organizations, such as the US Centers for Disease Control and Prevention (CDC) Autism Case Training,^12^ and from notable pediatric healthcare organizations, such as the Boston Children's Hospital's Developmental Screening module.^13^

### Challenges to DBP practice and education

The practice of interdisciplinary medicine is becoming increasingly more challenging with the increased prevalence of disabilities,^14^ increasing the demand for care with a limited number of trained providers, and changing reimbursement models that increasingly value metrics. Historically, the fee-for-service (FFS) payment models between health care providers and health insurance companies have paid limited attention to health outcomes and tended to favor procedurally based specialties.^15^ DBP practice, characterized by inadequate opportunities for revenue-generating procedures, engagement in non-traditionally billable activities (phone calls, chart reviews, electronic messaging, school notes, care coordination) and longer visit lengths (with fewer patients seen as a result) is historically not reimbursed well in a FFS model. Additionally, clinical productivity is often measured using benchmarks with target expectations^16^ mostly derived from DBP practice in academic settings that include time and effort expended on the education of trainees, research, and scholarly productivity. The increased emphasis on patient satisfaction and access to care^17^ creates a challenge for DBPs, given the relative paucity of practitioners, with patients traveling great distances to seek care,^18^ increasing referrals from primary care providers, and long wait lists.^19^

Despite a required 4-week concentrated educational experience in DBP (based on residency training guidelines), many pediatric residents miss instructional time, either being assigned to other perceived more urgent activities or for discretionary activities. This leads to insufficient time commitment to master either normal or abnormal child development and behavior, and graduates report feeling unprepared to manage developmental and behavioral concerns in practice.^20^ There has been interest in increasing the exposure to DBP at undergraduate medical education (UME) level, though time constraints, lack of qualified faculty availability and inadequate resources are barriers to effective implementation.^21^

### Strategies to address the challenges

To succeed as a specialty, DBPs need to be involved in self-advocacy and engage in educational endeavors to increase visibility, viability and interdisciplinary collaboration.

### Self-advocacy

DBPs should educate the leaders of pediatric departments^22^ and payers^23^ about the interdisciplinary and unique nature of DBP, explaining how some of the benchmarks applied to General Pediatrics may not be applicable to DBP. These discussions should include the degree of adjustments made to revenue- generation expectations for those with funded research, contracts, or administrative time. DBPs need to understand compensation metrics to advocate for fair and equitable pay and recognize that some current parameters are not specific for the field due to the limited amount of DBP practice data. Involvement at the institutional, state and national level through active participation and engagement in professional organizations is helpful in this regard. DBPs are encouraged to use professional coders and perform intermittent chart audits to maximize billing. DBPs need to be aware of sources of patient satisfaction data, national benchmark database, and their performance expectations. Collaboration with educators can range from sharing information about scientific developments related to children and adolescents with specialized health care needs to stay current (and updating trainees) about relevant laws, local and state regulations pertaining to the delivery of healthcare to these children and adolescents.

### Trainee education

It is essential to educate DBP trainees about different models of care delivery and emphasize workflow efficiencies and the business aspects of medical practice, to prepare them for their future roles, both in practice and in academics. This should include practicing "just enough" documentation to meet patient care and regulatory needs, support advocacy, and enable billing and compliance.^24^ Research and professional networks can help identify innovative models of care that include treatment outcomes, patient satisfaction, as well as the cost and value chain.^25^ While some quality measures appropriate for DBP have been developed,^26^^,^^27^ future payment models will require the development of value metrics for the inadequately reimbursed activities such as report generation, records review, and care coordination. Including patients and families in the process of metric development help maintain a patient-centered perspective.

It is promising that residency and fellowship education is moving towards curricula that include Entrustable Professional Activities (EPAs), which are observable aspects of a professional's typical daily work. EPAs are used to assess when a trainee is considered competent to be entrusted with a particular activity. The level of entrustment progresses (or should progress) from novice to entirely competent, and from requiring full in-person supervision to no supervision. This framework places competencies into clinical contexts that make practical sense for the trainee, though this is yet to be implemented fully at residency and fellowship level.^28^ Increased emphasis on DBP awareness at the UME level can engage medical students through opportunities for participating in research projects of faculty, and by DBP faculty delivering lectures on basic disability awareness in the preclinical curriculum. Another of MCHB's programs, the Leadership Education in Neurodevelopmental and Related Disabilities (LEND) in collaboration with the Association of University Centers on Disabilities has online content that serve as a resource.^29^

To complement the limited time on the DBP concentrated educational experience, longitudinal educational experiences in the ambulatory setting will be helpful, especially if guided by faculty with experience and training in DBP. Due to the limited availability of DBPs in many training programs, innovation and collaboration will be key, not just in training models, but also in addressing access barriers for patient care. Models such as co-location^30^ and telemedicine,^31^ will be helpful. Innovations in funding strategies can supplement traditional funding; these include contract-based work with agencies as part of interdisciplinary teams (schools or health departments), pairing with revenue-rich pediatric and surgical subspecialties for interdisciplinary clinics, and seeking philanthropy from foundations. Trainees need to be educated about revenue models, by participating in billing and coding and discussions about alternate payment models.

### Collaboration

While patient access barriers are not immediately solvable with the limited number of DBPs, collaborative models can be sought that include integrated behavioral health, where DBPs partner and take a leadership role.^23^ Collaborative office rounds (COR)^32^ allow DBPs and other mental and behavioral health experts to continue the education of primary care pediatric providers in delivering local care that allows "closer to home" care for many families, can reduce access barriers to DBP, and help primary care colleagues increase their confidence and expertise in managing DBP problems. DBP educators can champion interdisciplinary practice with trainees by recognizing barriers to collaboration, such as time constraints, disagreement on diagnosis and treatment plans, different laws around information sharing, and engaging in advanced skill development to foster collaborative relationships.^33^ It is important to engage parents and families for feedback in improving aspects of DBP care. Additionally, longitudinal curricular design for medical students^34^ and increasing family participation in curriculum through innovations like flipped classroom designs are promising.^35^

## Conclusions

Although the evolution of the medical training and healthcare delivery poses several challenges to the field of DBP and its training models, there are opportunities to engage in collaboration and use innovative models in DBP education across the learning spectrum and continuum. New approaches to post-graduate medical education and increased engagement of undergraduate medical students, coupled with the use of online modules and collaborative approaches to practitioner education provide avenues for further exploration. Self-advocating by DBP clinician-educators will ensure visibility and viability for the field into a brighter future for the next generation of DBPs.

### Conflict of Interest

The authors declare that they have no conflict of interest.
